# Hemoglobin level and survival in cervical cancer with chemoradiotherapy at high altitude, 2020–2022

**DOI:** 10.3332/ecancer.2024.1767

**Published:** 2024-09-13

**Authors:** José Fernando Robles Díaz

**Affiliations:** Regional Institute of Neoplastic Diseases of the Center, Av Progreso N° 1235,1237 y 1239, Concepción, Junín 12125, Perú; l https://orcid.org/0000-0001-9319-9458

**Keywords:** altitude, hypoxia, cervical cancer, hemoglobin, survival, uterine cervix

## Abstract

**Background:**

The purpose of this retrospective study was to determine the prognosis of altitude and pre-treatment hemoglobin (Hb) levels with progression-free survival (PFS) among women from the jungle and Andean regions of Peru with cervical cancer (CC) receiving weekly cisplatin and concurrent radiotherapy followed by brachytherapy or teletherapy boost.

**Methods:**

Patients with advanced clinical stage II-IVA CC were grouped according to Hb level (≥ 12.0, 11.9–10.0, 9.9–9.0 and ≤ 8.9 g/dL). Outcome measures were PFS, overall survival and local PFS.

**Findings:**

Between 1/2020 and 12/2022, 159 patients contributed demographic, clinical, pre-treatment Hb and outcome data with a median follow-up of 38 months. Kaplan–Meier estimates for survivals according to pre-treatment Hb level were significant when compared to a level of ≤8.9 g/dL, while estimates with altitude did not show statistical significance. Cox regression analysis of PFS demonstrated that pre-treatment Hb levels ≤8.9 g/dL (*p* = 0.000) were a significant factor. Age (*p* = 0.023), stage (*p* = 0.000), tumour size (*p* = 0.006) and treatment duration (*p* = 0.000) were also significant in the regression model.

**Interpretation:**

There is no difference between altitude and survival, but the difference in pre-treatment Hb level was a prognostic indicator of survival, with a Hb level of ≤8.9 g/dL being the worst prognosis.

## Introduction

Globally, in 2020, an estimated 604,127 cases of cervical cancer (CC) and 341,831 deaths were reported, with an age-standardised incidence rate of 13.3 cases per 100,000 women per year and a mortality rate of 13.3 cases per 100,000 women per year [1]. Concurrent chemoradiotherapy containing cisplatin (CCRT) followed by brachytherapy (BT) is a standard for the medical care of patients with advanced-stage CC. Unfortunately, advances in local control and survival following the regimen have plateaued over the past two decades, particularly for those with advanced pelvic disease [2].

Anemia and tumour hypoxia are two clinical factors impacting strategies intensifying treatments aimed at improving local control and survival. A significant percentage of CC patients present hemoglobin (Hb) levels below 12.0 g/dL [3]. Most studies have shown that anemia before and during treatment predicts poorer local control and is associated with shorter survival outcomes. The mechanism appears to be related to tumour hypoxia driving resistance to CCRT while simultaneously stimulating angiogenesis [4, 5].

In Latin America, there is a large population living at high altitudes, clinically defined as altitudes ≥2,500 m above sea level, where physiological changes begin to appear [6]. This finding raises questions about whether we should follow recommendations to maintain Hb above 10.0 g/dL or whether a correction factor for Hb results should be used as in the pediatric population. Not all populations living at the same altitude have similar Hb values, as seen in the Himalayas, where Tibetans have lower Hb concentrations than Han ethnic people and high-altitude residents [6, 7]. This could be explained by the fact that Tibetans have lived in the highlands for more than 25,000 years, while Han people have lived there for no more than 70 years, but high-altitude residents have lived in the Andes region for at least 9,000 years [8]. There are no publications indicating whether altitude influences survival outcomes or if it is necessary to correct Hb according to WHO recommendations for populations living at high altitudes [7].

Considering these backgrounds, the current retrospective study was designed to investigate the prognostic factor of Hb level and altitude prior to treatment with clinical outcomes among patients with CC in the central region of the country.

## Methodology

### Population

The institutional ethics committee, located at 3,283 m above sea level, approved this retrospective study in 2023 [9]. The study population consisted of a sample of women over 18 years of age diagnosed with CC at stages IIB to IVA according to the International Federation of Gynecology and Obstetrics (FIGO 2021) between January 2020 and December 2022 [10] (Table 1). The study population resided in the central region of the country, ranging from the Andean region at over 2,000 m above sea level to the jungle region at less than 2,000 m above sea level [11]. From 233 attended women, a sample of 159 women was selected based on a type I error of 0.05 and a confidence level of 0.975. Patients in this study had histologies such as squamous or adenocarcinoma. Patients underwent CCRT followed by either BT or external beam radiotherapy. The minimum chemotherapy cycles were four with cisplatin. Patients were segregated into groups based on pre-treatment Hb levels of ≥12.0, 11.9–10.0, 9.9–9.0 or <9.0 g/dL. The institute provided all demographic, tumour, Hb and follow-up data. These data did not differentiate whether a patient received a blood transfusion to raise the Hb level above 9.0 g/dL before treatment.

### Treatment

External beam radiotherapy delivered 50–56 Gy in 25–28 fractions using conventional or intensity-modulated radiotherapy techniques. For cervical BT boost, high-dose BT (28 Gy in 4 fractions) was used for clinical stage IIB-IIIB, while for stage IVA, external beam radiotherapy of 20 Gy in 5 fractions was added using conventional 3D radiotherapy techniques [12]. The total radiation cycle duration was intended not to exceed 56 ± 3 days. Cisplatin (40 mg m^−2^, not exceeding 70 mg total per week) was administered intravenously once weekly for a maximum of six cycles. Modifications of cisplatin dose during external beam radiotherapy were allowed; however, there were no consistent guidelines for cisplatin dose modification among treating physicians during the study period.

### Statistical analysis

The primary endpoint of the current study was progression-free survival (PFS), with secondary endpoints of overall survival (OS) and local progression-free survival (LPFS). Progression was defined as clinical or imaging evidence of persistent, recurrent or metastatic disease post-therapy. PFS was determined by the interval between diagnosis and disease progression, death or until the date of last contact for censored events (i.e., those alive without evidence of disease). OS was defined as the time from diagnosis to death or date of last contact for censored events (i.e., those alive regardless of disease status). LPFS was considered successful if no disease developed within the irradiation portals. LPFS was considered a failure if there was disease progression or persistence after treatment within the irradiation portal. Overall, patients were followed up quarterly for 2 years, and then biannually for the following 3 years.

Survival estimates were calculated using the Kaplan–Meier method, and differences between Hb groups were compared using the log-rank test. Cox proportional hazards model was performed to evaluate the impact of Hb level and residential altitude on PFS, OS and LPFS, adjusting for prognostic factors of age and tumour size as continuous variables, while Eastern Cooperative Oncology Group (ECOG) performance status, disease stage and radiation duration were categorical variables. Univariate analyses of these prognostic factors and pre-treatment Hb levels were evaluated using the Mann–Whitney *U* test and Kruskal–Wallis test since the result in the Kolmogorov-Smirnov test was less than 0.00 (Table 2). A *p*-value less than 0.05 (two-tailed) determined statistical significance. Data analysis was performed using SPSS statistical software (version 25).

### Role of the funding source

The IREN Center participated in the collection, analysis and interpretation of data.

## Results

### Population characteristics

This retrospective study included 159 patients who received documented CCRT and had Hb levels available for review. The median follow-up was 38 months, and follow-up data were available for all 159 patients. Demographic, clinical and treatment data are presented in Table 1. Out of 159 patients, 127 (79.9%) resided at an altitude higher than 2,500 m above sea level and 136 (85.5%) had an ECOG of 0 or 1. The mean age was 54.7 ± 11.4 years. Patients had stage IIB (29.9%), stage III (59.8%) or stage IVA (10.4%) at the time of diagnosis. One hundred and fifty-one (95.0%) had a histopathological diagnosis of squamous cell carcinoma. The mean tumour size was 4.9 ± 1.8 cm. The mean duration of the entire regimen was 65.8 ± 11 days.

The mean pre-treatment Hb level was 13.2 ± 2.6 g/dL. One hundred and eleven (69.8%) had ≥12.0 g/dL, 20 (12.6%) had 11.9–10.0 g/dL, 14 (8.8%) had 9.9–9.0 g/dL and 14 (8.8%) had <9.0 g/dL. Blood transfusion prior to treatment to raise the Hb level above 9.0 g/dL was not reliably recorded in the available data. A pre-treatment Hb level of <12.0 g/dL was significantly associated with age, stage and histology at the time of diagnosis (Table 2, *p* < 0.05). Residential altitude was not significantly associated with any variables at the time of diagnosis.

### Survival

**Figure 1.**
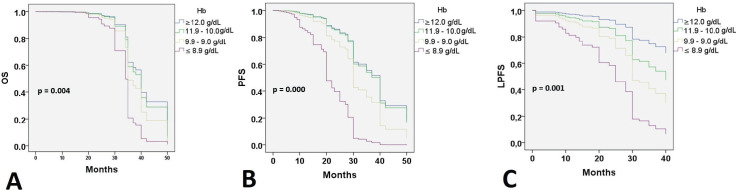
Survival according to the Hb level. OS: Overall survival; PFS: Progression-free survival; LPFS: Local progression-free survival; Hb: Hemoglobin level.

At 24 months of follow-up, 118 (74.2%) patients were alive with no evidence of disease; 20 patients were alive with disease progression, and 21 (13.2%) women had died. Twenty-six (11%) patients had failed in local control with CCRT. Thirty-five (22%) developed distant recurrences outside the irradiation field. Kaplan–Meier estimates for PFS, LPFS and OS according to pre-treatment Hb level were significant when compared to Hb level ≤8.9 g/dL (Figure 1), while estimates with altitude did not reach statistical significance.

Cox regression analysis of PFS showed that pre-treatment Hb levels of ≤8.9 g/dL (*p* = 0.000) were a significant factor in PFS. Age (*p* = 0.023), stage (*p* = 0.000), tumour size (*p* = 0.006) and regimen duration (*p* = 0.000) were also significant in the regression model. Cox regression analysis of LPFS demonstrated that pre-treatment Hb levels of ≤8.9 g/dL (*p* <0.000) were a significant factor in LPFS. Age (*p* = 0.019) and stage (*p* = 0.000) were also significant in the regression model. Cox regression analysis of OS showed that pre-treatment Hb levels of ≤8.9 (*p* = 0.002) were a significant factor. Age (*p* = 0.010), ECOG (*p* = 0.009), stage (*p* = 0.000) and regimen duration (*p* = 0.001) were also significant in the regression model (Table 3).

### Discussion

In CC patients, approximately 37% typically have Hb levels equal to or greater than 12.0 g/dL [3], unlike our study, which represents more than 69% of cases. This contradiction cannot be explained solely by clinical stage, as in our cases, voluminous tumour disease predominates, leading to blood loss. However, it can be explained by the influence of altitude, as the study population usually resides at high altitudes of 2,500–3,500 m above sea level (Table 1) [13].

This study was conducted to clarify whether altitude influences survival outcomes or if there is a need for correction of Hb values as occurs in the pediatric and pregnant populations [14]. In our univariate analyses, pre-treatment Hb levels less than 12.0 g/dL were not associated with altitude (Table 2). No Hb correction was performed, as Hb correction for high-altitude populations biases toward a higher prevalence of iron deficiency anemia, with uncorrected Hb being more accurate, particularly in populations above 3,500 m above sea level [14, 7].

Only cases at stage III/IVA at the time of diagnosis were more likely than stage II disease to have lower Hb levels before treatment. This is consistent with previous research, which has also reported that poor functional status and large tumour size (> 4 cm) are associated with low Hb levels in CC patients before treatment [3, 15]. The potential explanations for these associations are difficult to determine, but it is suggested that impoverished patients tend to have worse functional status, seek medical attention less frequently and present with bulky central pelvic disease or advanced clinical stage disease [16].

**Figure 2.**
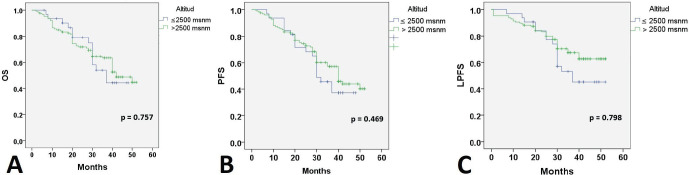
Survival according to altitude. OS: Overall survival; PFS: Progression-free survival; LPFS: Local progression-free survival; Hb: Hemoglobin level.

When searching for prognostic factors among demographic, clinical and Hb variables for PFS, OS and LPFS in CC, it was evident that altitude did not influence survival (Figure 1). Therefore, recommendations from clinical guidelines regarding minimum Hb values to initiate oncologic treatment in CC patients residing at less than 3,500 m above sea level should not be feared. It was found that for LPFS and PFS, both outcomes were contributed to by age, clinical stage (mainly IIIC2 and IVA), and Hb (≤ 8.9 g/dL). Except for the influence of tumour size and regimen duration, which only impacted PFS. Additionally, for OS, ECOG was added (Table 3). The three survival outcomes shared the marked influence of pre-treatment Hb when ≤8.9 g/dL regardless of altitude (Figure 2). This aligns with the study by Khamis *et al* [17] in Tanzania, where an Hb level above 9 g/dL had a higher OS after radiotherapy compared to those with an Hb level <9 g/dL. Our study did not capture information on Hb level during treatment; however, when adjusting for other prognostic factors, a pre-treatment Hb level ≤8.9 g/dL remained a significant contributing factor to worse survival. The underlying reasons for this association between anemia and poor outcomes remain difficult to understand in CC patients. An alternative hypothesis offers justification that large, hypervascular tumours or advanced clinical-stage tumours bleed more easily, which manifests as clinical anemia. Many consider an Hb level <10.0 g/dL to be an epiphenomenon associated with poor therapeutic outcomes [3, 15], and thus, transfusion aimed at correcting anemia is not expected to provide clinical benefits. For example, Bishop *et al* [18] published a retrospective study on over 2,400 patients with advanced clinical stage CC. The authors reviewed Hb levels and disease outcomes after radiotherapy and concluded that only disease-specific survival (HR = 1.49) was affected by Hb levels <10.0 g/dL. Their multivariate analysis did not identify an impact on central pelvic recurrence or distant metastasis. Furthermore, transfusion was associated with poor outcomes in all measures for the entire study cohort, although the mechanism of this is still unknown [18].

Clinical stage IIIC2 and IVA had the worst outcomes, likely because teletherapy was delivered over longer periods. In stage IIIC2, the pelvis was irradiated first, followed by the para-aortic segment, while in stage IVA, everything was irradiated with external beam radiotherapy due to the unavailability of a BT applicator. These technical deficiencies may have contributed to the risk being markedly higher in these clinical stages, which, in addition to primary tumour components, had nodal metastasis that did not receive simultaneous doses with the primary tumour [19].

The findings of our studies resolve the controversial issue of whether patients fare worse because they have anemia and live at altitude, as they have the same survival, but all patients will be affected if they start treatment with levels lower than 8.9 g/dL. Blood transfusion is unlikely to allow the patient to escape poor survival, so patients should be diagnosed with screening programs to prevent moderate anemia.

One limitation of the study is that it cannot be safely replicated in the older age population who are Quechua residents of southern Peru, as a lower Hb level has been observed than in central Peru residents, indicating greater adaptation to altitude [20]. Despite these limitations, our study also has strengths. First, to the best of our knowledge, this is the first prognostic factor study with Hb considering residential altitude, providing an overview of the literature in this area. Second, the study suggests that blood transfusion should not be recommended when levels are above 8.9 g/dL. Finally, we believe that our results can contribute to the scientific evidence on the prognostic value of pre-treatment Hb for iron deficiency diagnosis in high-altitude residents.

## Conclusion

There is no influence of altitude on Hb level or survival in CC in central Peru, so the Hb correction formula for altitude should not be used. If the oncologic regimen is initiated with an Hb level ≤8.9 g/dL, worse survival outcomes are likely. Prospective studies on Hb levels and other hematologic markers before and during treatment should be conducted.

## Conflicts of interest

There is no conflict of interest.

## Figures and Tables

**Table 1.** Demographic, clinical and treatment characteristics (*n* = 159).

Variable	Number	Mean	SD	%
Age (years)	159	54.7	11.4	
< 50	49			30.8
50–65	78			49.1
≥66	32			20.1
Altitude (msnm)		2,682	1,111.8	
< 1,000	28			17.6
1,000–2,499	4			2.5
2,500–3,500	116			73.0
>3,500	11			6.9
ECOG				
0–1	113			71.1
2–3	46			28.9
Histological type				
Squamous	151			95.0
Adenocarcinoma	8			5.0
Clinical stage				
IIB	47			29.9
IIIA	0			0.0
IIIB	12			7.4
IIIC1	60			38.1
IIIC2	23			14.3
IVA	17			10.4
Tumour size, cm		4.9	1.8	
≤4.0	66			41.5
>4.0	93			58.5
Duration,^¥^ days		65.8	11.8	
<60	55			34.6
≥60	104			65.4
Hb, g/dL		13.2	2.6	
≥12	111			69.8
11.9–10.0	20			12.6
9.9–9.0	14			8.8
≤8.9	14			8.8

¥Regimen duration: If stage IIB to IIIC1, involve the time for CCRT and BT. If IIIC2, involves the time for CCRT-BT plus para-aortic radiotherapy. If IVA, involves the time for CCRT plus radiotherapy to residual cervical tumour

**Table 2.** Association between Hb level (≥12 versus ≤11.9 g/dL) with demographic, clinical and treatment characteristics (*n* = 159).

Variable	Average rank	*p* value
Age (years)^*^		0.001
< 50	93.32	
50–65	78.42	
≥ 66	63.45	
Altitude^*^		0.181
< 1,000	73.04	
1,000–2,500	115.63	
2,501–3,500	80.67	
> 3,500	77.68	
ECOG^£^		0.237
0–1	77.8	
2–3	85.4	
Histological type^£^		0.000
Squamous	77.6	
Adenocarcinoma	125.6	
Clinical stage^*^		0.002
IIB	62.8	
IIIB	95.8	
IIIC1	82.5	
IIIC2	94.0	
IVA	88.7	
Tumour size, cm^£^		0.707
≤4.0	81.3	
>4.0	79.1	
Duration,^¥^ days^£^		0.827
<60	79.1	
≥60	80.5	

¥ Treatment duration: If it is stage IIB to IIIB, it involves the time for CCRT and BT. If it is IIIC2, it involves the time for CCRT-BT plus para-aortic radiotherapy. If it is IVA, it involves the time for CCRT plus radiotherapy to the residual tumour of the cervix

£ Mann–Whitney *U* test (*p* < 0.05, statistically significant)

* Kruskal–Wallis test was used (*p* < 0.05, statistically significant)

**Table 3.** Multivariable analysis for survival outcomes (*n* = 159)

Variable	OS				PFS				LPFS			
	*p*	HR	IC		*p*	HR	IC		*p*	HR	IC	
			LI	LS			LI	LS			LI	LS
Age	0.010	1.038	1.009	1.067	0.023	1.028	1.004	1.053	0.019	1.037	1.006	1.070
Altitude	0.962	1.000	1.000	1.000	0.518	1.000	10.000	10.000	0.967	1.000	1.000	1.000
ECOG	0.009	2.260	1.224	4.173	0.047	1.870	0.993	3.254	0.078	1.778	0.938	3.369
Histological Type	0.876	0.903	0.252	3.241	0.830	1.153	0.314	4.235	0.253	1.290	0.116	1.764
Clinical Stage	0.000				0.000				0.000			
IIIB	0.893	1.104	0.262	4.649	0.462	1.542	0.487	4.886	0.086	7.591	0.752	76.596
IIIC1	0.027	2.650	1.117	6.285	0.002	3.185	1.521	6.666	0.001	28.324	3.789	211.751
IIIC2	0.000	13.266	3.361	52.364	0.001	5.797	2.045	16.438	0.003	27.340	3.121	239.466
IVA	0.000	399.060	56.463	2820.414	0.000	47.366	14.999	149.578	0.000	207.857	21.240	2,034.129
Tumour size	0.742	1.102	0.620	1.958	0.006	2.080	1.233	3.509	0.384	1.298	0.721	2.338
Duration	0.001	4.030	1.798	9.031	0.004	2.582	1.346	4.955	0.057	2.014	0.979	4.141
Hb, g/dL	0.023				0.000				0.000			
11.9–10.0	0.549	1.306	0.546	3.122	0.706	1.159	0.539	2.493	0.306	1.554	0.668	3.613
9.9–9.0	0.449	1.379	0.600	3.170	0.130	1.786	0.843	3.788	0.117	1.940	0.847	4.445
≤8.9	0.002	4.137	1.660	10.308	0.000	9.198	3.330	25.404	0.000	10.140	3.494	29.431

A *p*-value <0.05 is statistically significant. OS: Overall survival; PFS: Progression-free survival; LPFS: Local progression-free survival; HR: Hazard ratio; CI: 95% Confidence interval; LL: Lower limit; UL: Upper limit
